# A Case Report of Tissue Mosaicism in 45,X0/46,XY: Diagnostic Complexity in a Newborn with Ambiguous Genitalia

**DOI:** 10.3390/reports8030146

**Published:** 2025-08-15

**Authors:** Mariola Krzyścin, Agnieszka Brodowska, Dominika Pietrzyk, Katarzyna Zając, Elżbieta Sowińska-Przepiera

**Affiliations:** 1 Pediatric, Adolescent Gynecology Clinic, Department of Gynecology, Endocrinology and Gynecological Oncology, Pomeranian Medical University in Szczecin, ul. Unii Lubelskiej 1, 71-252 Szczecin, Poland; krzyscin@o2.pl (M.K.);; 2 Department of Gynecology, Endocrinology and Gynecological Oncology, Pomeranian Medical University in Szczecin, Unii Lubelskiej 1, 71-252 Szczecin, Poland; 3 Individual Laboratory for Endocrine Diagnostics, Pomeranian Medical University in Szczecin, ul. Unii Lubelskiej 1, 71-252 Szczecin, Poland

**Keywords:** Turner syndrome, sex determination, delayed puberty, mosaic karyotype, case report

## Abstract

**Background and Clinical Significance**: The 45,X0/46,XY mosaic karyotype is categorized as a disorder of sex development and can lead to atypical sexual development. Latent mosaicism involving Y chromosomal segments may be much more prevalent than previously assumed, according to a growing number of findings. This primarily depends on how sensitive cytogenetic methods are—such as traditional karyotype screening, FISH methods, or molecular analyses. **Case Presentation**: We present the case of a 10-week-old infant with hermaphroditic external genitalia. During pregnancy, ultrasonography revealed severe fetal development difficulties, including severe widespread edema. An abnormal 45,X0/46,XY mosaic karyotype was discovered during a genetic amniocentesis conducted during the 16th week of pregnancy. The infant was born in average general condition at 39 + 6 weeks of gestation. Physical examination of the infant revealed features of facial dysmorphia, webbed neck, and hermaphroditic external genitalia. The testicle was palpable on the left side, but the gonad was absent on the right. Laboratory tests revealed a typical hormonal profile of the mini-puberty period in boys. Moreover, a hormone panel and thyroid ultrasound were performed; congenital hypothyroidism was diagnosed. Three separate independent sources of biological material were used in cytogenetic analysis to determine the karyotype: skin fibroblasts (to confirm tissue mosaicism), oral epithelial cells (FISH), and peripheral blood lymphocytes. It showed that a mosaic occurred very early in embryogenesis by confirming the existence of karyotypes 45,X and 46,XY in various tissues (mosaic tissue distribution). **Conclusions**: Tissue mosaicism should be compared to the analysis of tissues from other embryonic origins, including blood and oral tissue. Support for gender identity and treatment decisions, including the prediction of the future risk of gonadoblastoma, as well as multidisciplinary care, is necessary.

## 1. Introduction and Clinical Significance

Sex determination, a fundamental aspect of human biology, usually occurs based on the presence of the Sex-determining Region Y (SRY) gene, which initiates the development of the testes and the male phenotype in a Y chromosome-positive population [1]. However, the presence of chromosome mosaicism, involving the coexistence of cells with different genetic material, can lead to atypical sexual development [2]. An unusual puberty can happen for this reason, which also presents significant diagnostic and therapeutic decision-making difficulties [3]. However, there is a wide range of phenotypes associated with sex development abnormalities, and patients are frequently not easily classified with a single diagnosis [4]. The 45,X0/46,XY mosaic karyotype is one such complicated example, according to the Chicago Consensus on the Management of Intersexuality. It is classified as a disorder of sex development (DSD) [5]. Defined as congenital conditions in which the development of chromosomal, gonadal, or anatomic sex is atypical, differences or disorders of sex development (DSDs) encompass a range of distinct diagnoses, some of which are described and grouped in Table 1 below. The effects of the various DSD variants on the interior and exterior reproductive organs vary widely [6]. Many factors influence a patient’s development, including gender differences in brain structure, genes related to sexual maturation, prenatal exposure to androgens, and cultural factors [7].

Turner syndrome, one of the most prevalent chromosomal diseases, affects around 1 in 2500 female births [8]. The typical karyotype of 45,X0 corresponds to the classic female phenotype, with characteristics such as short stature, webbed neck, heart defects, and hypergonadotropic hypogonadism, leading to infertility and delayed puberty [9]. Approximately 5% of Turner syndrome cases are found to have genetic material of the Y chromosome, which is associated with an increased risk of gonadal neoplasia, especially the development of gonadoblastoma [10]. However, an increasing number of reports indicate that latent mosaicism involving Y chromosome fragments may occur much more frequently than previously thought, which largely depends on the sensitivity of the cytogenetic method used, such as classical karyotype screening, FISH techniques, or molecular analyses [11]. Males, people with hermaphrodite genitalia, and girls with Turner syndrome are only a few of the many phenotypes that can result from the 45,X0/46,XY mosaicism. Diagnostically and therapeutically, these cases are particularly complicated due to the variability in clinical presentation and the ambiguity that results from chromosomal, gonadal, and phenotypic sex determination [12]. They require a multidisciplinary approach that considers both medical (hormonal, cytogenetic, diagnostic imaging), as well as psychological and ethical aspects [13].

The case of an infant with a 45,X0/46,XY mosaic karyotype and ambiguous external genitalia is discussed in this study, with an emphasis on the challenge of cytogenetic, imaging, and hormonal diagnosis, as well as the gender determination issues in the context of tissue mosaicism.

## 2. Case Presentation

A 10-week-old infant with abnormal genital development was brought by her parents to the Pediatric and Adolescent Gynecology Outpatient Clinic in Szczecin for genital evaluation. The infant, formally registered by the parents as a girl, showed features of an ambiguous genital structure.

**Figure 1:**
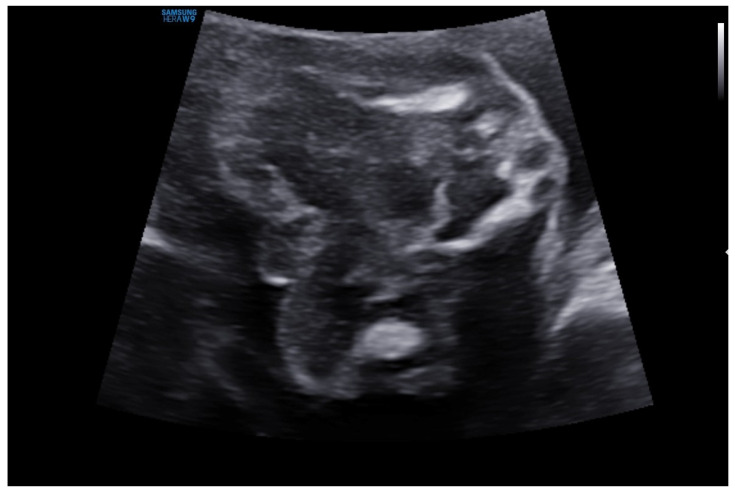
Ambiguous genitalia observed during an ultrasound examination at 20 weeks of pregnancy.

From the parents’ reports and the documentation provided, the mother’s pregnancy showed significant abnormalities of fetal development from the first prenatal examination in the form of intense generalized edema (head, neck, lungs), shortened limbs, and swelling of the head and nuchal fold. Prenatal testing in the first trimester of pregnancy identified a high risk of trisomy 21. Ultrasound examination revealed fetal edema, NT 3.5 mm. In the 16th week of pregnancy, a genetic amniocentesis was performed, and an abnormal 45,X0/46,XY mosaic karyotype was found. In the 20th week of pregnancy, ambiguous genitalia were observed during an ultrasound examination, as presented in Figure 1.

Due to significant fetal edema and a suspected heart defect, an extremely unfavorable prognosis for fetal survival was presented. After a detailed explanation of the risks, the parents decided to continue the pregnancy. In subsequent ultrasound examinations, the fetal edema became less and less severe. At 39 + 6 weeks of gestation, the baby from her first pregnancy was eventually delivered in a tiny town at the lowest referral hospital. Because labor was not progressing, a transperitoneal suprapubic caesarean section was performed. The baby was born in average general condition, with a weight of 2900 g, length 53 cm, and received 6, 8, 10, and 10 points on the Apgar scale.

**Figure 2:**
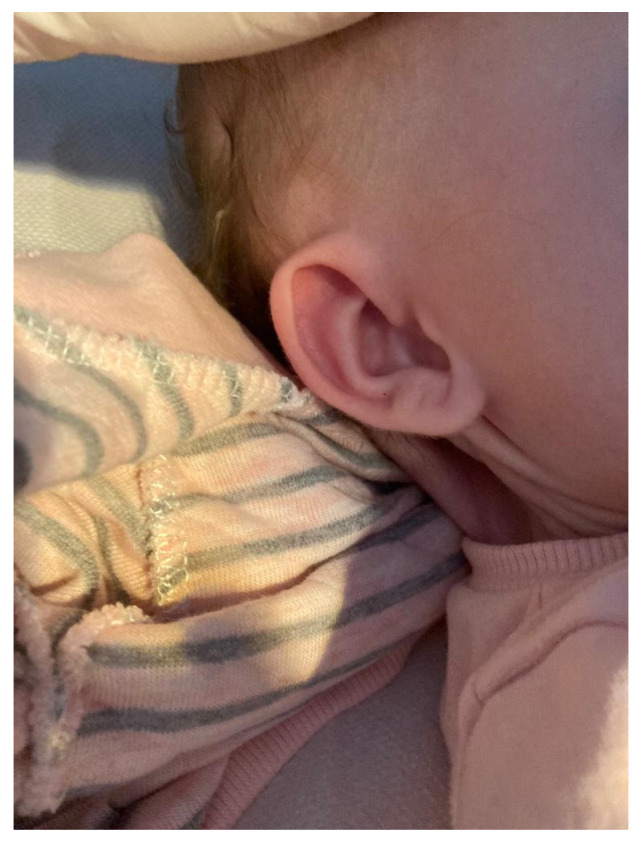
Webbed neck.

After birth, physical examination of the deviants revealed features of facial dysmorphia, a ribbon-shaped neck, as presented in Figure 2.

**Figure 3:**
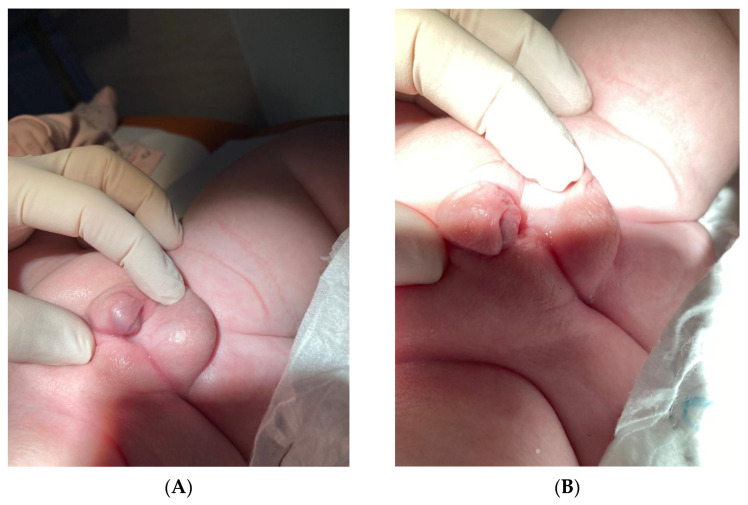
Atypical external genitalia. (**A**) Left side scrotum with a palpable gonad. Right labioscrotum without a gonad. (**B**) The urethral outlet located at the base of the phallic process, which is estimated to be 2 cm long and 1.3 cm wide.

Other abnormalities in the appearance of the infant were hermaphroditic external genitalia. The appearance of the hermaphroditic external genitalia was documented in Figure 3A,B.

The scrotum was found on the left side with a palpable testis, while on the right side, there was no palpable gonad and incomplete differentiation of the genital fold. The urethral orifice was located at the base of a structurally enlarged penis 2 cm long and 1.3 cm wide, with an appearance reminiscent of spodziectasia. In the first 10 weeks of age, the newborn had no functional problems with the urogenital system apart from external appearance abnormalities. The newborn urinates through a micropenis.

Laboratory tests performed during the first week of life showed elevated testosterone levels (1.21 ng/mL) and a predominance of LH over FSH, consistent with the typical hormonal profile of the mini-puberty period in boys. AMH levels were depressed (low level is normal for males), and estradiol was below the threshold of quantification. Screening results for congenital adrenal hyperplasia (17-OH-progesterone) were normal. Table 2 summarizes the biochemical blood tests performed in the newborn on day 7 of life.

The infant had persistently elevated TSH levels (28–33 uIU/mL) with normal values of free thyroid hormones. This was the reason for an ultrasound of the thyroid gland—it showed reduced parenchymal echogenicity and a heterogeneous echostructure without clear focal changes. Based on this, congenital hypothyroidism was diagnosed, and treatment with levothyroxine was initiated. Table 3 shows the laboratory and ultrasound results of the thyroid gland.

The infant underwent a pelvic ultrasound; no ovaries or uterus were visualized, and the testicle and epididymis were visible in the scrotal sac on the left side testicle, with normal echogenicity and echostructure, size 12 × 6 × 8 mm (volume 0.6 mL), an increased amount of fluid in the scrotal sac with a layer width of up to 8 mm, and epididymis without palpable changes. On the right side in the upper part of the inguinal canal was a tissue area of total size 13 × 9 × 4 mm, and a testis with epididymis with dysplasia features. MRI confirmed the absence of Müllerian structures and the presence of a right dysplastic testicle. Echocardiography showed benign coarctation of the aorta.

Cytogenetic analysis was performed to determine the karyotype using three different independent sources of biological material: peripheral blood lymphocytes, oral epithelial cells (FISH), and fibroblasts from the skin (to confirm tissue mosaicism).

1. Peripheral blood-classical analysis (GTG):

Karyotype extracted from peripheral blood lymphocyte culture (20 metaphases) showed mosaic:

1.1. Ten cells (50%): karyotype 45,X;
1.2. Six cells (30%): karyotype 46,XY with the presence of a translocation of a fragment of the short arm of the Y chromosome (Yp11.3) to another autosomal chromosome;
1.3. Four cells (20%): karyotype containing an unidentified chromosomal marker (mar), not included in the classical nomenclature.

2. FISH (fluorescence in situ hybridization)-buccal epithelial cells:

FISH analysis using centromere probes X (DXZ1), Y (DYZ3), and the SRY probe (Yp11.3) showed the following:

2.1. A total of 46% of cells contained only a signal for the X centromere;
2.2. A total of 48% of cells contained signals for both X and Y, with the presence of the SRY gene;
2.3. A total of 6% of cells showed the presence of a signal for a marker chromosome with undetermined SRY status.

In all, the presence of the SRY gene was demonstrated in approximately 50% of the cells assessed by FISH.

3. Skin fibroblasts:

A neck fold biopsy was performed, obtaining a similar mosaic pattern, which confirmed the presence of karyotypes 45,X and 46,XY in different tissues (mosaic tissue distribution), indicating a mosaic formed very early in embryogenesis.

The parents were informed about the complexity of the clinical situation and the possible consequences—the risk of infertility, the risk of neoplasia from dysgenetic gonads, and the need for their removal, as well as the possibility of future genital corrective surgery and gender identity issues. After a joint consultation of the gynecology–urology–endocrinology team, it was considered that the child should be raised as a boy at this stage, which was presented to the parents as a proposal, with the indication that the final decision remains with them. However, the child’s parents unanimously decided to register the child as a girl, as this is what their intuition tells them.

At this stage, further diagnostic imaging was planned—urography and MRI of the abdomen and pelvis, as well as possible qualification for surgery to remove the dysgenetic right gonad and biopsy of the left gonad. Currently, there is no universally agreed-upon treatment strategy. Confirmation of abnormalities based on physical examination and chromosomal analysis is necessary before deciding on treatment and further management in order to fully understand the risk of malignancy and fertility potential. In the discussion, potential diagnostic and therapeutic options based on gonadectomy or periodic follow-up examinations in low-risk individuals are described in more detail.

The parents and the child received psychological and endocrinological care with follow-up of sexual development and gender identity in the following months and years of life.

## 3. Discussion

The presented case report illustrates the complexity of diagnosis and treatment of a newborn with unclear genital development and a mosaic karyotype of 45,X0/46,XY. A prenatal diagnosis was concluded early in the pregnancy based on abnormal ultrasound exams of the fetus, which showed shortened long bones and edema. A 45,X0 and a 46,XY cell line containing the SRY gene were observed in a culture of fibroblasts from amniotic fluid, indicating a mosaic karyotype with characteristics that direct male development and elements of Turner syndrome [14].

After delivery, classical cytogenetic analysis (GTG) from peripheral blood lymphocyte cultures revealed three cell lines: 45,X0 (50%), 46,XY with translocation of a Yp fragment (30%), and cells with an unidentified chromosomal marker (20%). The GTG (G-banding by trypsin with Giemsa) technique is the primary method in classical cytogenetics for karyotype analysis. If mosaicism is suspected, whether it is demonstrated in the karyotype, it is recommended that additional metaphases be analyzed or that more sensitive studies, such as in situ hybridization FISH and PCR [11], be performed. In this instance, the FISH technique was applied using probes for the SRY gene, the Y heterochromatin region, and the centromere of the X chromosome. This allowed for the confirmation that roughly 50% of the assessed oral epithelial cells expressed the SRY gene. Additionally, research revealed that 46% of cells had one copy of the X chromosome and 48% had both sex chromosomes, indicating a unique chromosomal mosaic with varying Y material concentrations among the different cell lines.

Due to the limited diagnostic value of analyzing only peripheral blood lymphocytes, a skin biopsy was taken to culture fibroblasts. Confirmation of tissue mosaicism was reached, suggesting a very early origin of the disorder in embryogenesis. When mosaicism is suspected, the varied distribution of cell lineages in tissues with distinct germline origins (mesoderm-lymphocytes, ectoderm-oral epithelium, and mesoderm-gonads) is consistent with recent studies that show the necessity of karyotype evaluation on several tissue types [15].

On physical examination, the infant appeared to have a mixed phenotype, with the presence of a scrotum on the left side with a testicle and partial differentiation on the right side. Hypospadias was indicated by the urethral outflow on the underside of the enlarged penis. The hormonal profile (elevated testosterone, predominance of LH over FSH) was consistent with the physiological “mini puberty” in males. However, reduced AMH levels and the absence of Müllerian structures and the presence of only one well-developed gonad indicated partial gonadal dysgenesis [16]. The data confirm the presence of the mosaic 45,X0/46,XY and identify atypical cell lines with chromosome marker and Yp fragment translocation. The SRY gene and gonadal determinant genes (such as SOX9) may exhibit irregular expression because of these modifications, which might be responsible for partial masculinization and a mixed phenotype [17]. The presence of the SRY gene in about half of the cells may be consistent with the development of testicular tissue on the left side and dysgenesis on the right side. Intratissue variation in mosaicism also explains the phenotypic features of Turner syndrome coexisting with partial virilization [18].

Patients with different sexual development (DSD), particularly those with Y chromosome material, are more likely to develop gonadal germ cell cancers (GCTs). Gonadoblastoma is the most frequent tumor observed in this patient population, and some estimates suggest that there is a 15–35% chance of developing GCTs.

It has been suggested that tumor formation in DSD patients is caused by a combination of OCT3/4 and TSPY expression in germ cells [19]. Risk factors also include the role of gonadotropins, particularly the follicle-stimulating hormone (FSH), estrogens, and androgens, which are responsible for a higher risk of cancer, especially during puberty. Based on the histopathological examination of the gonadal biopsy sample, a diagnosis of precancerous change can be made. However, this risk varies greatly based on a wide range of circumstances, making the choice to have a preventive gonadectomy quite difficult [5]. Nevertheless, since the peripheral aromatization of residual testicular testosterone produces estradiol, delaying gonadectomy until at least adolescence permits natural pubertal development. By postponing the procedure, the patient is able to take advantage of puberty’s physiological results, which can be highly significant for their general development and mental health [20].

However, the actual risk of malicious intent is difficult to determine. This mosaicism can lead to a wide range of sexual development, from typically male to female or ambiguous [21]. Particularly when it comes to irreversible surgical procedures, physicians should wait until a patient is mature enough and capable of making their own decisions. As a result, there is no universally agreed-upon treatment strategy. Therefore, clinicians must consider individual risk and choose the treatment strategy that is most appropriate for the patient, which can be assessed over time through regular follow-up visits and examinations [19].

The described disorders have important implications not only for sex determination and genital development but also for the neurobiology of sex differentiation. The brain—as an organ subject to sexual differentiation—responds to genetic, hormonal, and epigenetic signals already in fetal life [22,23,24]. Expression of the SRY gene and its effect on SOX9 activation is not only consistent with testicular development but may also affect central nervous system structures responsible for gender identity formation, such as the hypothalamus, nucleus bearing striatum (BNST), amygdala, and substantia nigra [25]. However, in cases of the mosaic karyotype, the presence of the SRY gene may be restricted to specific tissues—such as the gonads, but not the brain—which can lead to a disparity between the physical phenotype and neurobiological sex identification. Deregulation of the expression of other genes, such as DAX1 (NR0B1) located on the X chromosome, whose overexpression acts antagonistically to SOX9, can further disrupt brain differentiation, even in the presence of functional testes [26]. Regardless of the presence of gonads, experimental research has demonstrated that modifying the SOX9/DAX1 expression ratio can change the process of sexual neurodifferentiation [27,28].

Also, the hormonal environment during the prenatal period is crucial for brain differentiation. Patients with partial gonadal dysgenesis often have irregular and delayed pulses of testosterone, as well as enzymatic disorders (for example, 5α-reductase or aromatase) that limit the conversion of testosterone to dihydrotestosterone (DHT) or estradiol [29]. Estradiol in the brain has an important masculinizing function, especially during critical periods of differentiation [30,31,32]. Disruption of these hormone levels can result in feminization of brain structures despite the presence of androgens [33].

**Figure 4:**
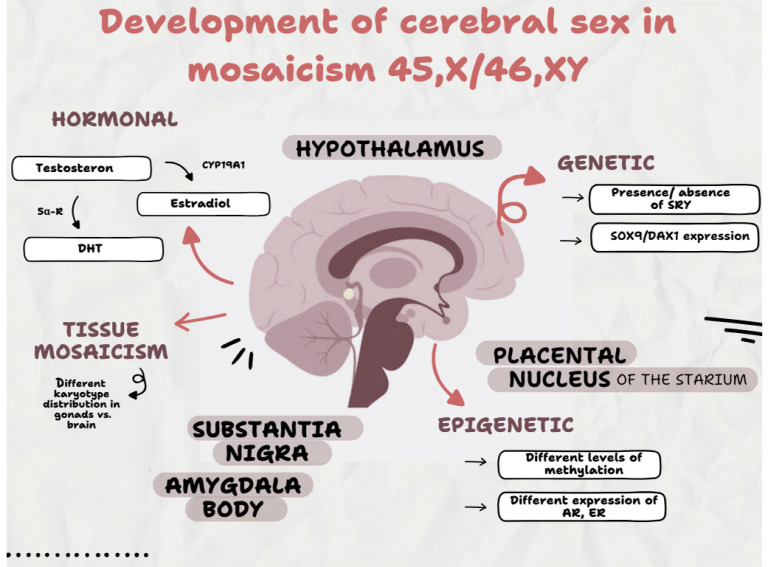
Development of cerebral sex in mosaicism 45,X0/46,XY. A schematic illustration of how hormones, genetic material, and epigenetic changes affect brain tissue development in relation to the human body’s sexual and gender identity development.

A variable distribution of 45,X0 and 46,XY cells between tissues is also seen in karyotypic mosaicism cases. This means that the distribution of cells containing the Y chromosome (and thus the SRY gene) may be significantly different between the gonad and brain, which could lead to future incompatibility between assigned sex, gender identity, and the development of secondary sex characteristics during adolescence [34,35]. Moreover, the presence of chromosomal markers of unknown origin can epigenetically affect neuronal differentiation pathways through abnormal methylation of sex gene promoters [36]. The scheme (Figure 4) illustrates the effects of the hormones, genes, and epigenetic changes described in the text above on the brain’s development of sexual identity.

In conclusion, a patient with a mosaic karyotype of 45,X/46,XY, even with the presence of functional testes and testosterone production in the neonatal period, remains at increased risk of developing gender identity incompatibility and possible gender dysphoria in the future [37]. One meta-analysis showed that the overall prevalence of gender dysphoria is approximately 15% among adolescents and adults with DSD [38]. Therefore, the decision on gender assignment should be made with the greatest care—preferably by an interdisciplinary team—taking into account the future possibilities of neuropsychological assessment, gender identification, and the family’s readiness to support the child’s long-term psychosocial development.

In countries like Poland, whose laws only allow for a binary classification of sex into female and male, the situation is more problematic. Due to these restrictions, parents are obligated to make decisions regarding their children’s gender assignment based on insufficient knowledge about the development of their future gender identity, which adds to their difficult circumstances [39,40]. The lack of formal recognition of neutral or indeterminate gender can lead to difficult socio-legal situations and pressure for quick decisions, which in the long run may not be appropriate to the patient’s actual psychosocial development.

## 4. Conclusions

As previously stated, a variety of phenotypes are present in 45,X0/46,XY mosaicism, which can impact growth, fertility, gonadal development, and hormonal balance. Because tissue mosaicism varies, this should be combined with an examination of tissues from various embryonic origins, such as blood and oral tissue, to forecast the risk of gonadoblastoma in the future. Multidisciplinary care is required, as is support for gender identification and treatment choices. The diagnosis represents a diagnostic and therapeutic challenge, requiring an individual approach with medical, psychological, and ethical considerations.

## Figures and Tables

**Table 1:** Classification of sex development disorders (DSDs).

(**A**)			
**Group**	**Subcategory**	**Examples**	**Key Features/Cause**
Disorders of Gonadal Development	- Gonadal dysgenesis (complete/partial) - Ovotesticular DSD	- Swyer syndrome (46,XY complete gonadal dysgenesis) - 46,XX/XY ovotesticular DSD	Defects in genes regulating early gonad formation (e.g., SRY, NR5A1, DAX1)
Disorders of Androgen Synthesis or Action	- Defects in testosterone or DHT production - Androgen insensitivity syndromes	- 5α-reductase deficiency - Complete/partial AIS	Normal gonads (testes) but impaired masculinization of external genitalia
Disorders of Gonadal Hormone Excess	- Androgen excess in XX individuals - Aromatase deficiency	- Congenital adrenal hyperplasia (CAH) - Maternal virilization	Excessive androgen action during fetal development
Syndromic and Complex DSD	- Multisystem syndromes involving sex development	- Campomelic dysplasia (SOX9 mutation) - Denys–Drash syndrome (WT1)	DSD is part of broader genetic syndrome affecting multiple organs
Chromosomal Mosaicism or Chimerism	- Sex chromosome mosaicism or mixoploidy	45,X/46,XY mosaicism 46,XX/46,XY chimerism	Discordant development due to mixed cell lines; variable phenotype
Unclassified/VUS (Variant of Uncertain Significance)	- Unknown or novel genetic cause	- Gene panel detects VUS - No clear correlation yet	Requires further research and individualized assessment
(**B**)			
**Karyotype**	**Category**	**Example Conditions**	**Key Features**
46,XX	XX DSD (ovarian)	- Congenital adrenal hyperplasia (CAH)	- Typical ovaries - Androgen excess may cause virilization
46,XY	XY DSD (testicular)	- Androgen insensitivity syndrome (AIS) - 5α-reductase deficiency	- Gonads are testes - Variable external genitalia - Often undervirilization
Sex Chromosome DSD	Sex chromosome anomalies	- Turner syndrome (45,X) - Klinefelter syndrome (47,XXY) - Mixed gonadal dysgenesis	- Gonadal dysgenesis - Atypical development of genitalia or secondary sex traits

**Table 2:** Biochemical parameters of the patient on the 7th day of life.

Parameters	Value
Testosterone	1.21 ng/mL
FSH	2.1 IU/L
LH	4.3 IU/L
AMH	14.6 ng/mL
Estradiol	<5 pg/mL
17-OH-Progesterone	36 ng/dL

Biochemical parameters of the patient: FSH, follicle-stimulating hormone; LH, luteinizing hormone; and AMH, Anti-Müllerian Hormone.

**Table 3:** The laboratory and ultrasound results of the thyroid gland.

Parameters	Result
TSH	28–33 uIU/mL
Free T4	1.1 ng/dL
Free T3	3.0 pg/mL
Thyroid ultrasound	Hypoechogenic, heterogenous gland

Biochemical parameters of the patient: TSH, *thyrotropic hormone*; Free T4, *free thyroxine*; and Free T3, *Free triiodothyronine*.

## Data Availability

The original contributions presented in this study are included in the article; further inquiries can be directed to the corresponding authors.
